# Trace element and metal sequestration in vitellaria and sclerites, and reactive oxygen intermediates in a freshwater monogenean, *Paradiplozoon ichthyoxanthon*

**DOI:** 10.1371/journal.pone.0177558

**Published:** 2017-05-12

**Authors:** Beric M. Gilbert, Annemariè Avenant-Oldewage

**Affiliations:** Department of Zoology, University of Johannesburg, Johannesburg, Gauteng, South Africa; University of Pretoria, SOUTH AFRICA

## Abstract

Exposure to metals and other trace elements negatively affects infection dynamics of monogeneans, including diplozoids, but, physiological mechanisms linked to exposure have yet to be documented. In this study sequestration of trace elements and reactive oxygen intermediate production in the monogenean, *Paradiplozoon ichthyoxanthon*, was demonstrated. During dissection of host fish, *Labeobarbus aeneus*, the gills were excised and assessed for *P*. *ichthyoxanthon*, which were removed and frozen for fluorescence microscopy or fixed for transmission electron microscopy. Trace elements were sequestered in the vitellaria and sclerites in *P*. *ichthyoxanthon*, and the presence of reactive oxygen intermediates was observed predominantly in the tegument of the parasite. Trace elements and metals identified and ranked according to weight percentages (wt%) in the vitellaria were Cu > C > Au > O > Cr > Fe > Si while for the sclerites C > Cu > O > Au > Fe > Cr > Si were identified. For most element detected, readings were higher in the vitellaria than the sclerites, except for C and O which were higher in sclerites. Specifically for metals, all levels detected in the vitellaria were greater than in sclerites. Based on the proportion of trace elements present in the vitellaria and sclerites it appears that most trace elements including metals were sequestered in the vitellaria. The results of reactive oxygen intermediate production in the tegument of the parasite suggests either trace element accumulation takes place across the tegument or results from the action of the host’s immune response on the parasite. The results serve as the first demonstration of trace element sequestration and reactive oxygen intermediates in a freshwater monogenean parasite.

## Introduction

Aquatic organisms are exposed to and accumulate a wide variety of naturally occurring trace elements which become stored in inert tissues and subcellular compartments. Toxic effects associated with accumulated metals and some trace elements become evident in organisms when these elements bind inappropriately to sensitive organelles and molecules [1–3]. To control toxic effects associated with the accumulation of trace elements, biota have evolved mechanisms to minimize and regulate the interaction of reactive trace elements with proteins and other cellular components, and optimally utilise essential elements [4]. This is achieved through binding to metalloproteins which have an affinity for binding metals and other trace elements and effectively decrease excess levels of trace elements. Vitelloproteins, are such proteins, have been found to be useful biomarkers of exposure in invertebrates and have been shown to bind a number of trace elements [5–7]. In this way, the levels of free trace elements including metals which can potentially form free radicals within cells are reduced [8].

Trace element accumulation in parasites and their use as bioindicators in aquatic ecosystems has received increased attention [9]. Most of these investigations have analysed metals and other trace element concentrations in endohelminths of fishes, particularly, cestodes [10–15], acanthocephalans [16–19], nematodes [20–25] and digeneans [26]. Of the groups studied, acanthocephalans demonstrate the highest accumulation potential followed by cestodes. However, little is understood about the fate of metals and trace elements in parasites. Studies on *Bothriocephalus scorpii* and *Schyzocotyle* (formerly *Bothriocephalus*) *acheilognathi* [27] indicated that gravid, egg laden proglottids accumulate trace elements to higher levels compared to the anterior mature and immature proglottids [10,28]. Similarly in the acanthocephalan, *Moniliformis moniliformis*, metal burdens in female worms have been shown to be higher than in males [29]. For both cestodes and acanthocephalans, this was related to the egg shells functioning as sequestration sites for trace element including metals [10,30,31].

Exposure of parasites to metals and metalloids has also been shown to result in elevated stress responses or biomarker responses in parasites. Exposure to arsenic (As^III^) and antimony (Sb^III^) was found to result in cell death of *Leishmania* spp. but through different mechanisms [32]. Palladium exposure resulted in elevated production of heat shock proteins in the cysticanths [33] of *Polymorphus minutus*.

Globally, metal and trace element accumulation studies in ectoparasites have lagged behind those for endoparasites, and have mostly focused on the effects at a population level [34–37]. Laboratory studies on gyrodactylids [38–42] and larvae of *Paradiplozoon ichthyoxanthon* [43] documented sensitivity toward exposure to a number of metals. The exact mechanism by which these metals exert their toxic effects on the parasites studied is still largely unknown. The aims of this study were therefore to determine where in the body of adults and diporpas of *P*. *ichthyoxanthon* metals are sequestered and which metals are present at these sites in adult worms, and to document events of oxidative stress though visualisation of reactive oxygen intermediates in adult parasites, which may be due to metals becoming accumulated.

## Materials and methods

### Parasite collection

During a single survey to the Vaal Dam, South Africa, in winter (August 2014), *Paradiplozoon ichthyoxanthon* specimens were collected from *Labeobarbus aeneus*. The host fish were caught by means of gill nets (mesh size: 45–190 mm) around UJ Island (26°52’33.62”S; 28°10’25.76”E) and 36 live fish were removed from the nets, in accordance to permits from the Gauteng Department of Agriculture for collection of fish (permit number: CPE3000123). Live fish were transported back to a field laboratory and maintained in aerated live wells containing dam water. Thereafter, fish were weighed, measured and euthanized by spinal severance posterior to the head. All procedures involving animals in this study were done in accordance with ethics guidelines as set out by the South African National Animal Ethics Council and approval by the University of Johannesburg Ethics committee (Protocol number: 9 April 2013). The gills were excised and assessed for *P*. *ichthyoxanthon*. Seventy seven parasites were removed from the gills and 38 were flash frozen in liquid nitrogen while the remainder were fixed in 2.5% glutaraldehyde in 1 M sodium cacodylate buffer for 1 hour and then post fixed in 1% OsO_4_ (w/v) in 1 M sodium cacodylate buffer (pH 7.2) at 4°C.

### Cryomicrotomy and fluorescence microscopy

Whole mounts and sections of flash frozen parasites were prepared for fluorescence microscopy. Microscope slides were cleaned in acidic alcohol and dried at 60°C and coated with poly–L–lysine (Sigma-Aldrich, Missouri, United States of America). A working solution of poly–L–lysine was prepared by diluting 100 mL stock with 1000 mL deionized water, and used to coat slides that were dried over night at room temperature. Cryosections of 10 frozen *P*. *ichthyoxanthon* were prepared by embedding whole worms in optimal cutting temperature (OCT) compound (Sakura Finetek, California, United States of America) and freezing in a bath of dry ice and 2-methyl butane (Sigma-Aldrich, Missouri, United States of America) until the compound turned white [44]. The blocks were removed and stored at -80°C until sectioning on a Reichert—Jung CryoCut E cryomicrotome at 5 μm. Sections were mounted on the coated slides, air dried for 60 minutes and stored at -80°C until staining.

The fluorochromes used for visualising metal ions and reactive oxygen intermediates (ROI) in sections were Phen-Green^™^ FL cell-permant diacetate (Molecular Probes, Eugene, Oregon) and CellROX^®^ deep red (Molecular Probes, Eugene, Oregon) respectively. NucBlue (Molecular Probes, Eugene, Oregon), a derivative of DAPI, was used as a counter stain to visualise nucleic acids. Working solutions of the fluorochromes were prepared by dissolving 1 mg Phen-Green in 1 mL dimethyl sulphoxide (Merck, South Africa) and then diluting 5 μL of the solution in 495 μL milli-Q water. CellROX deep red stock solution was diluted to 5 μM in the Phen-green solution. Finally 50 μL of NucBlue stock solution was added to the Phen-Green—CellROX mixture and stored at 4°C until treatment of sections. Slides were removed from the freezer and air dried for 60 minutes beneath a UV lamp to photobleach. The sections were then mounted in the fluorochrome mixture, covered with a coverslip, sealed with clear nail varnish and incubated in the dark at room temperature for 30 minutes. Whole mounts of adult parasites and diporpas were similarly prepared by photobleaching, and mounting in the fluorochrome mixture. Slides were sealed with clear nail varnish. Sections and whole mounts were studied with a Zeiss Axioplan 2 epifluorescence microscope operated with Axiovision 4.3 software.

### Ultra-microtomey and EDS analysis

Ten paired adult parasites were separated at the fusion region and each individual in the pair were then post fixed in osmium tetroxide and then washed three times in fresh 1 M sodium cacodylate buffer (pH 7.2) for 10 minutes each. Thereafter specimens were dehydrated through an ascending series of ethanol (70, 80, 90, 96 and 100% for 15 minutes each) concentrations, infiltrated and embedded in Spurr’s resin. Resin blocks were polymerized in an oven for two days at 60°C. Semi-thin sections (70 nm) of *P*. *ichthyoxanthon* were made using a Reichter-Jung Ultracut E ultramicrotome, mounted on formavar coated copper grids and contrasted using 5% (w/v) uranyl acetate (Agar Scientific, Essex, United Kingdom) and lead citrate. Sections were studied and x-ray microanalysis was performed using a Jeol JEM 2100 transmission electron microscope (TEM) equipped with an 80 T X-Max^N^ SSD energy-dispersive x-ray spectrometer (Oxford Instruments, City, Country), operated at 200 kV in spot size 1 mode. Concentrations of trace elements and metals determined at the points in sections where scans were performed re expressed as normalised concentrations in weight percentage (wt%) of specific elements according to the setup of the instrument. The weight percentage is therefore a measure of the number of atoms at the analysis point in the section and accounts for the atomic mass of the specific elements detected. As osmium, lead and uranium are incorporated during preparation of samples for analysis these elements were included in the scans of sections but have been excluded from the results as they have no bearing on the levels of elements which are incorporated into adult parasites.

## Results

**Fig 1:**
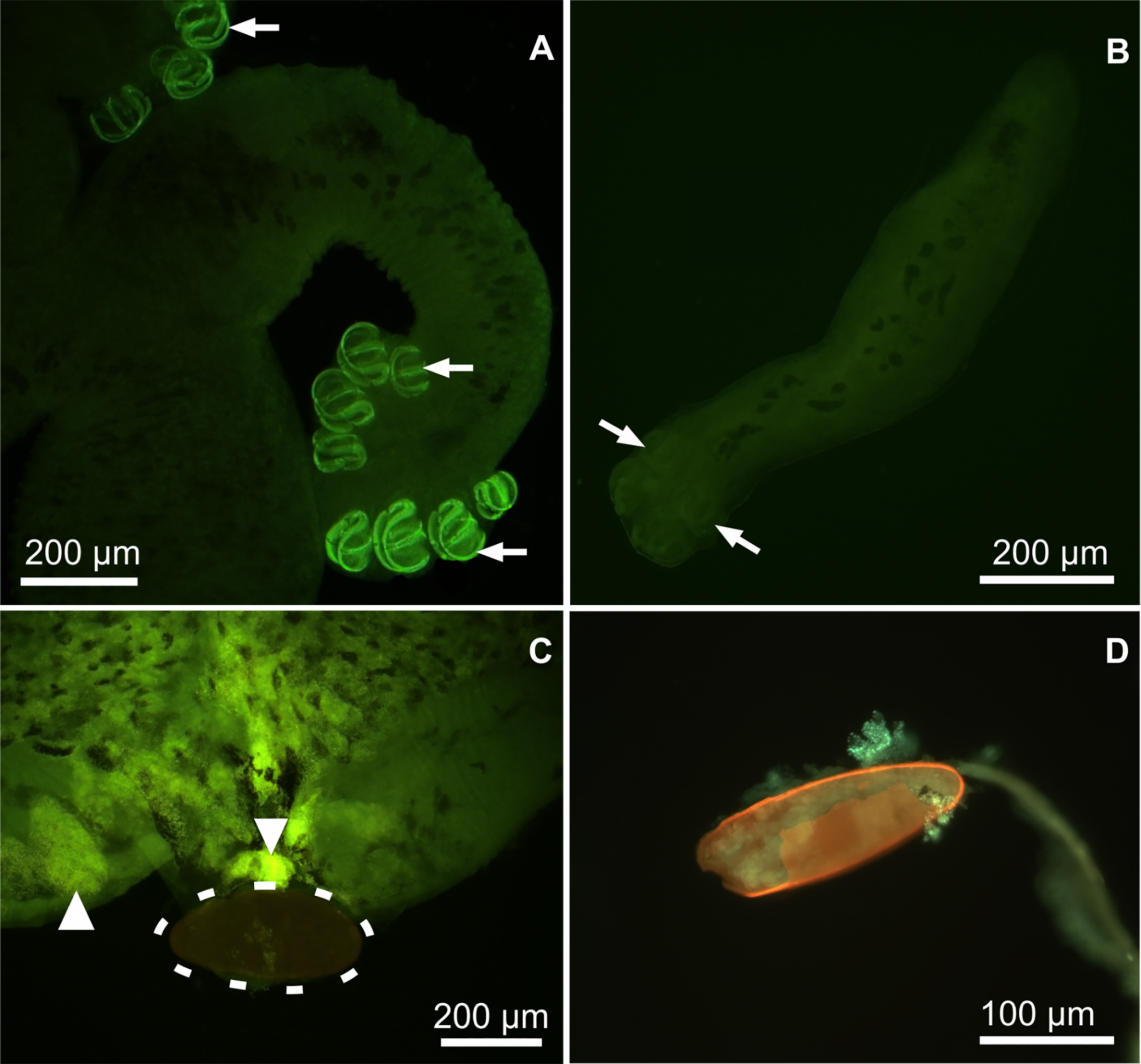
Micrographs showing whole mount of an adult (A and C) and diporpa (B), and a sectioned egg (D) of *Paradiplozoon ichthyoxanthon* stained only with Phen-Green. Sclerites of the clamps of the adult parasite and diporpa (white arrows) and vitellaria (white triangle) can be seen fluorescing, whereas, negative reaction to the fluorochrome by an in utero egg (encircled by dotted line) can be seen situated between posterior regions of the two individuals.

In whole mounts of adults and diporpas of *P*. *ichthyoxanthon* the presence of metals was confirmed by use of Phen-Green (Fig 1A–1C). In adult parasites, the sclerites of the clamps were found to fluoresce a bright green colour, compared to the body of the worms. This green fluorescence is indicative of the presence of metals, and the intensity correlates with the concentration within different body compartments [45]. Furthermore, the fluorescence of clamp sclerites of the adults was brighter when compared to diporpas. In adults, the vitellaria produced an intensely bright green fluorescence (Fig 1C and 1D) while the egg showed a negative reaction for the fluorochrome, as seen by the brown colour. Vitellaria were absent in diporpas.

**Fig 2:**
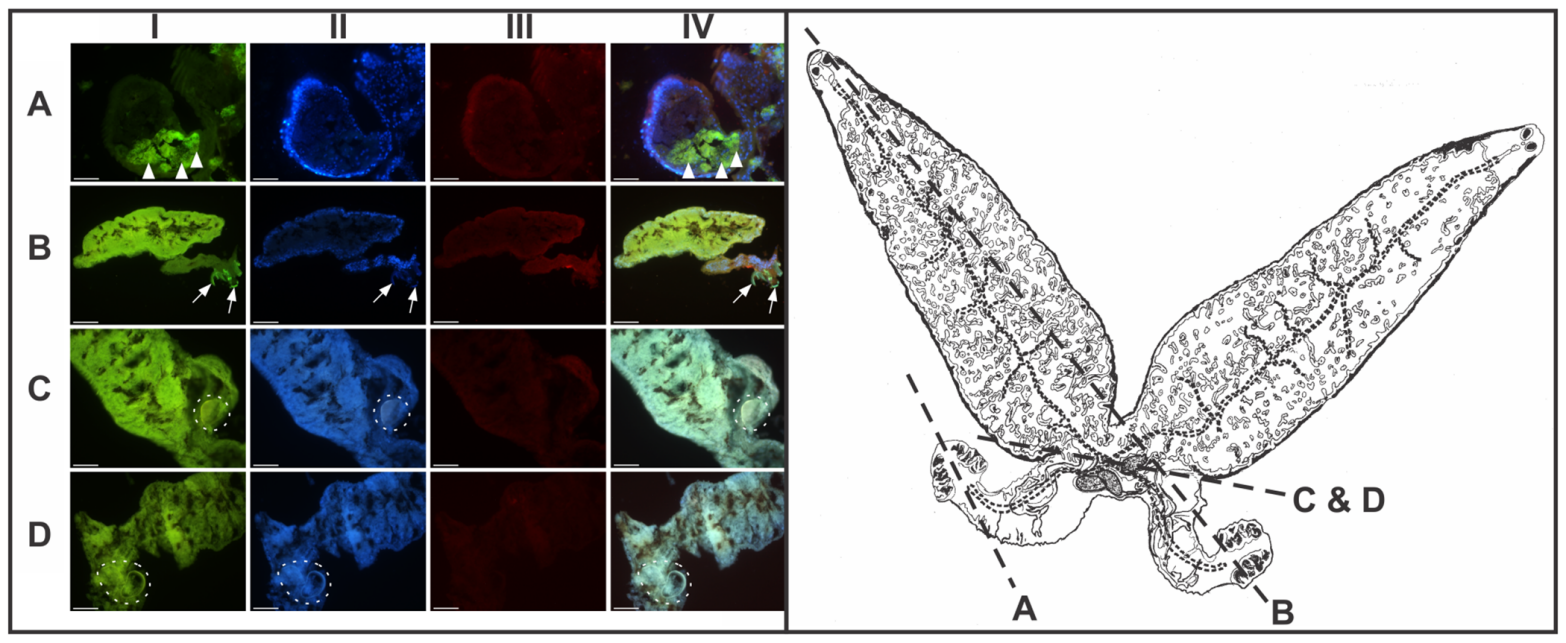
Micrographs showing sections through the haptor (A), longitudinal section of the anterior and posterior regions (B) and longitudinal sections through the fusion area and egg of *Paradiplozoon ichthyoxanthon* (C and D) stained with Phen-Green (I), NucBlue (II) and CellROX (III), and the combined image (IV). Triangles indicate the vitellaria, arrows the sclerites and sections through in utero eggs are encircled by a dotted line. Sections planes are indicated on the accompanying sketch of an adult parasite.

Results of the fluorescence microscopy of sections of *P*. *ichthyoxanthon* are presented in Fig 2 and section planes are indicated in the line diagram of an adult *P*. *ichthyoxanthon*. Fluorescence micrographs of sections through adult *P*. *ichthyoxanthon* indicate positive reactions for metals and reactive oxygen intermediates. Positive reactions for metals are indicated by green fluorescence (I) and show clear internal partitioning of metals within the body of the parasite. Bright green fluorescence can be seen for the vitellaria (Fig 2A) and sclerites (Fig 2B), whereas, a weaker reaction for metals occurred for the tegument (Fig 2A) and egg shell (Fig 2C and 2D) of the parasite. Blue fluorescence indicates the presence of nuclear material within sections (II). Nuclei appear to be aggregated along the periphery of the organism (ie. within the tegument) and to a lesser extent around viscera. Reactive oxygen intermediate production is indicated by red fluorescence (III) and was identified in all sections through *P*. *ichthyoxanthon*. Brighter ROI fluorescence was found for the tegument, while weaker reactions could be seen for the visceral organs and parenchyma, and where bright fluorescence for metals was present. Differences in production of ROI were further noted for the anterior and posterior regions of the parasites. Bright fluorescence was noted in the posterior aspect of the parasites compared to the anterior, this is particularly evident by localised fluorescence of ROI in the longitudinal section through the anterior and posterior regions of the parasite (Fig 2B). Furthermore, areas where metals appear to be accumulated do not indicate a correspondingly intense fluorescence for ROI.

Identification of metals at sites in parasites where bright fluorescence was identified, was achieved through analysis of semi-thin sections by EDS. Although sections were contrasted using lead citrate and uranyl acetate it was possible to identify elements which did not have overlapping excitation wavelengths with elements in contrasting solutions. In both vitellaria and sclerites C, O, Si, Cr, Fe, Cu, and Au were detected (Table 1). Comparison of the detectable levels of the metals between vitellaria and sclerites of adult *P*. *ichthyoxanthon* differed, with higher element percentages detected in vitellaria compared to the sclerites. This was especially apparent when comparing the levels of metals (Cr, Fe, Cu, and Au), which were present in both organs. According to the weight percentages of the metals detected, in the vitellaria Cu was highest (44.49 wt%) and was followed by Au > Cr > Fe > Si. For the sclerites, all metals detected were lower than in the vitellaria and Cu (24.41 wt%) was similarly highest, followed by Au > Fe > Cr > Si being lowest (0.21 wt%).

**Table 1:** Weight percentages (wt%) for trace elements and metals detected at points within in the sclerites and vitellaria of *Paradiplozoon ichthyoxanthon* from the Vaal Dam, South Africa.

	Sclerites n = 20		Vitellaria n = 20	
	Mean (wt%)	Standard deviation	Mean (wt%)	Standard deviation
**Carbon**	49.95	21.70	28.20	23.47
**Oxygen**	3.79	2.14	1.34	1.10
**Silicon**	0.21	0.23	0.19	0.13
**Chromium**	0.43	0.51	1.24	0.80
**Iron**	0.45	0.39	0.61	0.47
**Copper**	24.41	16.52	44.49	18.06
**Gold**	2.48	4.04	5.66	3.36

n—number of observations in sections of adult *P*. *ichthyoxanthon*

## Discussion

This study indicates that trace elements accumulated by adults of *P*. *ichthyoxanthon* differentially bind to organs, and particularly the vitellaria and sclerites. X-ray analysis of the element composition of the vitellaria and sclerites of *P*. *ichthyoxanthon* indicated elements such as C, O, Si, Cr, Fe, Cu and Au. Comparison of the amount of each element detected in the vitellaria and sclerites indicates more metals and trace elements are sequestered to the vitellaria than the sclerites, except for carbon and oxygen which were higher in sclerites. Comparability of the element values between the sclerites and the vitellaria was possible due to the fact that structures were sectioned and mounted on copper grids. The point where readings were taken was done in the centre of the grids so as to further minimise the interference with the copper wire forming the grids. This meant that the sections were the same thickness and therefore interference from background levels of elements was the same for both structures. The interference of background levels with carbon stubs and sample thickness in x-ray analysis was also indicated by Shinn et al. [46] in a study on the element composition of the sclerotised haptoral elements in three species of *Gyrodactylus*. The differences in element values between the sclerites and the vitellaria further indicates that vitellaria has greater binding capacity for metals compared to the sclerites. The sequestration of metals to vitelline has been shown to be an effective mechanism for regulating the levels of trace metals in the adult organism [47]. This however, may have further implications with regard to the development of embryos as these may become transferred to developing juvenile parasites via the yolk from adults. Erasmus [48] further indicated that antimony in vitellaria of *Schistosoma mansoni* was strongly associated with the vitelline droplets in vitelline cells. Vitellogenin has further been shown to be a suitable biomarker for monitoring exposure to pollutants, such as metals [49,50] and from the current results the same may be possible for *P*. *ichthyoxanthon*. However, from the lack of fluorescence of the eggs, it appears that metals are not associated with the contents of the egg. This needs further clarification as there is no information regarding the vitelline ultrastructure in diplozoids. Other studies have documented similarities in ultrastructural development of vitelline cells between other monogeneans [51,52] and some digeneans [48,51].

Terrestrial and aquatic invertebrates sequester metals to the exoskeleton which is shed during ecdysis and thus functions as a possible method of regulating metals in the body [53–57]. In monogeneans, Shinn et al. [46] indicated vanadium was incorporated into the ventral bar of *Gyrodactylus caledoniensis* and the hamuli of *G*. *salaris* and *G*. *colemanensis*. Shinn et al. [46] indicated a high sulphur content in the sclerites of *G*. *caledoniensis*, *G*. *salaris* and *G*. *colemanensis*. The high affinity of trace elements including metal ions for SH–groups has been indicated in a number of proteins, particularly the metallothioneins which have been extensively studied in the role of trace element detoxification organisms. Kritsky et al. [58] indicated through staining the haptor of *Gyrodactylus* with Gomori’s trichrome, the ventral bar is composed of collagen while the composition of the hamuli comprise another protein which did not react with the stain. Lyons [59] and Shinn et al. [60] suggested that the sclerotized haptoral elements of gyrodactylids are composed of keratin–like proteins. Regarding the protein composition of the sclerites of diplozoids, two studies have suggested that these structures are composed of either chitin [61] or resilin–like proteins [62], which is similar to the composition of arthropod exoskeletons. Terrestrial and marine marcoinvertebrates have been shown to incorporate elements into their exoskeletons as a means of detoxification [63–65] and structural support [53–56]. As the clamps of diplozoids are possibly similar to the composition of arthropod exoskeletons, it is likely that elements incorporated into the sclerites may serve a similar structural supportive purpose. Element incorporation into the sclerites of *P*. *ichthyoxanthon* is evident from comparison of the intensity of the fluorescence of the clamps in adult parasites and diporpas. Such fluorescence intensity variance is indicative that the amount of metal and trace elements present in these structures [45] and variations between the developmental stages of the parasite could further relate to the length of exposure of the parasite. Schofield et al. [53] indicated that the amount of Zn present in the mandibles of leaf cutter ants differed between different developmental stages.

Negative fluorescence of the egg shell of *P*. *ichthyoxanthon* for metals was indicated and contrasts the results found for cestodes. In endoparasites, Sures et al. [10], Scheef et al. [29], Degger et al. [30] and Khalil et al. [31] collectively found that the eggs of the cestodes *B*. *scorpii* and *S*. *acheilognathi* function as sequestration sites for metals. Scheef et al. [27] suggested that metals were sequestered in eggs of the acanthocephalan, *M*. *monoliformis*, as female parasites contained greater metal concentrations than males. Sures et al. [10] found that the concentration of Pb and Cd were higher in the posterior, egg containing segments of *B*. *scorpii* compared to the anterior ones. Degger et al. [28] indicated that metals were associated with the egg shells of *S*. *acheilognathi* with the use of Phen-Green stain. Degger et al. [30] attributed negative results for metals in shells of *S*. *acheilognathi* to the age of the eggs, with younger eggs which did not contained metals and therefore producing a similar orange fluorescence as found in the present study for eggs of *P*. *ichthyoxanthon*. The negative result obtained in the present study can be linked to differences in the composition and formation of cestode and monogenean egg shells. According to Ramalingam [66] the monogenean egg shell is composed of dityrosine stabilised by sulphur cross-linkages and shells are not formed through a tanning process which occurs in cestodes [67], as they found that quinones were absent in egg shells of the monogeneans, *Pseudomicrocotyle* and *Pricea multae* after incubation with heated catechol [62]. The hardening of monogenean eggs is, therefore, achieved through dehydration of the shell matrix and not enzymatic polymerisation [68]. Metals have also been shown to sequester to the egg cases of sharks and skates [69,70], which are composed of similar proteins and harden via quinone tanning as in cestodes. It is thought that metals are incorporated into the shell matrix of skates, rays, dogfish sharks and cestodes during the formation of the egg shell.

The production of ROI by *P*. *ichthyoxanthon* was observed and localised predominantly to the tegument of the parasite, particularly in the region of the haptor. The production of ROI in relation of metals has been studied in a number of organisms, from protozoans to vertebrates. Exposure to environmental pollution has been found to produce biomarker responses in parasites. Mehta and Shaha [32] reported that exposure of *L*. *donovani* to As^III^ and Sb^III^ resulted in increased cell death due to oxidative stress. With accumulation of the metalloids resulting in an influx of Ca^2+^ ions and unavailability of glutathione (GSH) in reducing ROI in the cell due to the metals binding to the sulfhydryl groups of the protein. Not only did *P*. *ichthyoxanthon* produce ROI but this was localised to the tegument of the haptor. Sures and Radszuweit [33] found that exposure to elevated levels of Pd resulted in increased levels of heat shock protein (Hsp) 70 in *P*. *minutus* cysticanths infecting the haemocoel of *Gammarus roeseli*. Furthermore, the production of Hsp70 in response to Pd exposure was higher in the acanthocephalan cysticanths than both uninfected and parasitised gammarid intermediate hosts. Rico et al. [71] indicated localized production of ROI in certain areas of the cytoplasm and organelles of four ciliate species exposed to Cd and Cu. Even though it was shown that *P*. *ichthyoxanthon* produce ROI, it is unclear if this is specifically related to the accumulation of metals. The fact that bright ROI signals were localised in the posterior of the parasite does not necessarily indicated that production is related to the uptake of metals and other trace elements, but rather may be related to the interaction of the host immune response toward the parasite. Further investigation into this aspect is required and should constitute exposure of the parasites to metals under laboratory conditions.

## Supporting information

S1 Table Raw data of EDS scans for sections of parasites analysed by TEM. The data can be accessed from the following citation: The data supporting the conclusions drawn in this article can be accessed via Gilbert, Beric; Avenant-Oldewage, Annemariè (2017): EDS raw data. figshare. https://doi.org/10.6084/m9.figshare.4733320
(DOCX)
