# Supplementary material for: Trace element and metal sequestration in vitellaria and sclerites, and reactive oxygen intermediates in a freshwater monogenean, Paradiplozoon ichthyoxanthon
Source: PLoS One. 2017 May 12;12(5):e0177558. doi: 10.1371/journal.pone.0177558 (PMC5428946; doi:10.1371/journal.pone.0177558)
Supplement: S1 Table — The data can be accessed from the following citation: The data supporting the conclusions drawn in this article can be accessed via Gilbert, Beric; Avenant-Oldewage, Annemariè (2017): EDS raw data. figshare. https://doi.org/10.6084/m9.figshare.4733320 (DOCX) [file pone.0177558.s001.docx]

**S1 Table. Raw data of EDS scan for vitellaria (Vit) and sclerites (Scl) sections of parasites analysed by TEM.** Grey shaded blocks indicate elements which were present in chemicals used during sample preparation for TEM. Dashes (-) indicate instances where elements were not detected in the sample. The data supporting the conclusions drawn in this article can be accessed via Gilbert, Beric; Avenant-Oldewage, Annemariè (2017): EDS raw data. figshare. <https://doi.org/10.6084/m9.figshare.4733320>

| Specimen | Organ | C(wt%) | O(wt%) | Si(wt%) | Cr(wt%) | Fe(wt%) | Cu(wt%) | Os(wt%) | Au(wt%) | Pb(wt%) | U(wt%) |
| --- | --- | --- | --- | --- | --- | --- | --- | --- | --- | --- | --- |
| 1a | Vit | 26.39 | 1.08 | 0.13 | 0.36 | 0.83 | 38.61 | 7.99 | 2.2 | 18.28 | 3.23 |
| 1b | Vit | 25.85 | 1.16 | 0.11 | 0.33 | 0.76 | 37.63 | 9.09 | 2.38 | 19.06 | 2.73 |
| 2a | Vit | 31.48 | 1.63 | 0.16 | 0.32 | 0.65 | 33.31 | 9.01 | 2.04 | 18.25 | 2.38 |
| 2b | Vit | 27.36 | 1.07 | 0.15 | 0.36 | 0.74 | 35.26 | 8.8 | 2.29 | 20.66 | 2.46 |
| 3a | Vit | 7.96 | 0.42 | 0.18 | 0.77 | 0.61 | 45.19 | 10.59 | 8.98 | 17.02 | 7.72 |
| 3b | Vit | 13.98 | 0.14 | 0.08 | 2.33 | - | 61.95 | 7.7 | 9.73 | 20.86 | 3.99 |
| 4a | Vit | 9.3 | 0.61 | - | 1.66 | 0.38 | 57.9 | 5.13 | 12.16 | 10.85 | 6.17 |
| 4b | Vit | 9.03 | 0.35 | 0.05 | 1.51 | 0.71 | 56.61 | 18.74 | 5.3 | 6.41 | 0.17 |
| 5a | Vit | 6.85 | 0.58 | 0.24 | 1.41 | 1.15 | 63.02 | 6.94 | 7.04 | 6.05 | 6.66 |
| 5b | Vit | 82.33 | 3.56 | 0.05 | 0.26 | - | 11.24 | 0.7 | 0.7 | 1.01 | 0.07 |
| 6a | Vit | 85.2 | 2.79 | 0.51 | - | 0.23 | 9.22 | 0.12 | 1.75 | 0.81 | - |
| 6b | Vit | 27.57 | 2.45 | 0.21 | 2.2 | 0.09 | 42.47 | 5.48 | 4.57 | 13.12 | 1.69 |
| 7a | Vit | 16.75 | 0.01 | 0.29 | 2.14 | 1.82 | 59.95 | 11.14 | 11.14 | 0.42 |  |
| 7b | Vit | - | - | - | - | - | - | - | - | - | - |
| 8a | Vit | 25.02 | 2.67 | 0.25 | 2.26 | 0.07 | 47.7 | 4.88 | 4.96 | 12.64 | - |
| 8b | Vit | 11.31 | 0.39 | 0.03 | 2.1 | 0.83 | 54.15 | 11.97 | 6.44 | 6.39 | 5.72 |
| 9a | Vit | 3.71 | 0.07 | 0.22 | 0.11 | - | 84.17 | 5.31 | 8.21 | 0.55 | - |
| 9b | Vit | 55.24 | 1.46 | 0.13 | 1.33 | - | 32.08 | 1.17 | 4.03 | 4.3 | 0.25 |
| 10a | Vit | 29.42 | 2.66 | - | 1.73 | 0.25 | 43.93 | 1.09 | 7.42 | 9.75 | 2.96 |
| 10b | Vit | 41.1 | 2.36 | 0.47 | 1.06 | 0.08 | 31.01 | 0.2 | 6.18 | 15.74 | 1.35 |
| 1a | Scl | 25.85 | 1.16 | 0.11 | 0.33 | 0.76 | 37.63 | 9.09 | 2.38 | 19.06 | 2.73 |
| 1b | Scl | 31.48 | 1.63 | 0.16 | 0.32 | 0.65 | 33.31 | 9.01 | 2.04 | 18.25 | 2.38 |
| 2a | Scl | 27.36 | 1.07 | 0.15 | 0.36 | 0.74 | 35.26 | 8.8 | 2.29 | 20.66 | 2.46 |
| 2b | Scl | 29.06 | 1.57 | 0.15 | 0.4 | 0.74 | 32.24 | 8.8 | 2.09 | 20.46 | 2.32 |
| 3a | Scl | 1.02 | 2.2 | 0.87 | 1.89 | 0.72 | 71.78 | 6.46 | 16 | 5.6 | - |
| 3b | Scl | 70.76 | 4.56 | - | - | - | 20.76 | 1.14 | - | 8.74 | - |
| 4a | Scl | 57.72 | 4.39 | - | 0.55 | 0.96 | 10.09 | 1.18 | 4.38 | 20.09 | 1.69 |
| 4b | Scl | 33.75 | 10.33 | - | 1.36 | 1.3 | 41.81 | - | - | 19.42 | 1.6 |
| 5a | Scl | 38.6 | 5.38 | 0.61 | 0.73 | 0.27 | 24.34 | 9.12 | - | 22.09 | 0.47 |
| 5b | Scl | 65.14 | 3.79 | 0.14 | 0.13 | 0.12 | 12.53 | 2.61 | 0.64 | 14.12 | 0.61 |
| 6a | Scl | 73.03 | 4.23 | 0.09 | 0.08 | 0.08 | 7.5 | 1.98 | 0.51 | 12.16 | 0.24 |
| 6b | Scl | 67.3 | 3.61 | 0.11 | 0.12 | 0.12 | 12.16 | 2.74 | 0.54 | 12.64 | 0.52 |
| 7a | Scl | 61.47 | 3.28 | 0.13 | 0.13 | 0.17 | 21.7 | 3.41 | 1.09 | 8.11 | 0.71 |
| 7b | Scl | - | - | - | - | - | - | - | - | - | - |
| 8a | Scl | 61.05 | 3.28 | 0.13 | 0.13 | 0.17 | 21.7 | 3.41 | 1.11 | 8.11 | 0.71 |
| 8b | Scl | 67.37 | 3.61 | 0.11 | 0.12 | 0.12 | 12.13 | 2.73 | 0.53 | 12.6 | 0.53 |
| 9a | Scl | 73.09 | 4.24 | 0.09 | 0.08 | 0.08 | 7.48 | 1.98 | 0.51 | 12.13 | 0.24 |
| 9b | Scl | 65.14 | 3.79 | 0.14 | 0.13 | 0.12 | 12.53 | 2.61 | 0.64 | 14.12 | 0.61 |
| 10a | Scl | - | - | - | - | - | - | - | - | - | - |
| 10b | Scl | - | - | - | - | - | - | - | - | - | - |
